# Maternal Healthcare Services Utilisation and Its Associated Risk Factors: A Pooled Study of 37 Low- and Middle-Income Countries

**DOI:** 10.3389/ijph.2023.1606288

**Published:** 2023-10-23

**Authors:** Hasibul Hasan Shanto, Md. Akib Al-Zubayer, Benojir Ahammed, Md. Alamgir Sarder, Syed Afroz Keramat, Rubayyat Hashmi, Rezwanul Haque, Khorshed Alam

**Affiliations:** ^1^ Statistics Discipline, Science, Engineering and Technology (SET) School, Khulna University, Khulna, Bangladesh; ^2^ Centre for Health Services Research, Faculty of Medicine, The University of Queensland, Brisbane, QLD, Australia; ^3^ Centre for Housing Research, The University of Adelaide, Adelaide, SA, Australia; ^4^ School of Business, University of Southern Queensland, Toowoomba, QLD, Australia; ^5^ Centre for Health Research, University of Southern Queensland, Toowoomba, QLD, Australia

**Keywords:** maternal healthcare utilisation, antenatal care, skilled birth attendant, postnatal care, low- and middle-income countries

## Abstract

**Objectives:** The utilisation of maternal healthcare services (MHS) can play an essential role in reducing maternal deaths. Thus, this study examines the prevalence and factors associated with MHS utilisation in 37 low-and-middle-income countries (LMICs).

**Methods:** A total of 264,123 women were obtained from the Demographic and Health Surveys of 37 LMICs. Multivariate logistic regression was performed to identify the factors associated with maternal healthcare services utilisation.

**Results:** Around one-third (33.7%) of the respondents properly utilise MHS among women of childbearing age. In the pooled sample, the odds of MHS utilisation were significantly higher with the increase in wealth index, women’s age, age at the first birth, and husband/partner’s education. Urban residence (AOR [adjusted odds ratio] = 1.56; 95% CI [confidence interval]: 1.49–1.64), women’s autonomy in healthcare decision-making (AOR = 1.19; 95% CI: 1.15–1.24) and media exposure (AOR = 1.70; 95% CI: 1.58–1.83) were found to be the strongest positive factors associated with utilisation of MHS. In contrast, larger family (AOR = 0.93; 95% CI: 0.91–0.96), and families with 7 or more children (AOR = 0.72; 95% CI: 0.68–0.77) were significantly negatively associated with MHS utilisation.

**Conclusion:** The utilisation of MHS highly varied in LMICs and the associated factors. Expanding the wealth status, education, age at first birth, mothers’ autonomy in healthcare decisions, and media exposure could be essential strategies for increasing the utilisation of MHS; however, country-specific programs should be considered in national policy discussions. There is a need to formulate policies and design maternal health services programs that target socially marginalised women.

## Introduction

The process of bringing a child into the world is beautiful yet crucial simultaneously. A woman requires proper care for her and the child at this part of life. Maternal healthcare is usually defined as women’s healthcare during pregnancy, childbirth, and care during the postpartum period [1]. Ensuring maternal healthcare is necessary for both mother and child’s good health [1]. The utilisation of maternal healthcare services (MHS) is a complex behavioural phenomenon that includes recommended number of antenatal care (ANC) visits, delivery of a child by the skilled birth attendant (SBA), and appropriate postnatal care (PNC) services [2]. These are important to identify the potential risks of pregnancy, ensure birth with skilled care, and improve the health of the mother and newborn [3, 4].

Globally, many mothers fail to arrange the utilisation of MHS [5, 6]. During the last two decades maternal mortality ratio dropped by about 38% worldwide [7]. WHO estimated in 2017 that global maternal mortality was 211 per 100,000 live births [7], much beyond the UN’s SDG target 3.1 of less than 70 maternal deaths per 100,000 live births, with no country exceeding 140 maternal deaths per 100,000 live births by 2030 [8]. Insufficient antenatal care (ANC) visits, lack of delivery by a skilled birth attendant (SBA), and inadequate/absence of postnatal care (PNC) services make achieving the goals challenging [9], and have been found primarily in many low- and middle-income countries [5, 6].

Previous studies showed that poor MHS utilisation among mothers was influenced by various demographic and socio-economic factors [10, 11]. Key factors such as mother’s education, age, employment status, number of children, wealth index, and access to media coverage were found to be associated with MHS utilisation [4, 12]. Different studies at the national level, such as in Indonesia, Liberia, India, Bangladesh, and Sudan, tried to observe and determine the MHS utilisation influencing factors and the nature of their relations [3, 9, 13–15]. However, an explicit focus on the low- and middle-income countries on this scale is limited. Thus, our study tried to clarify this part of the existing literature gap. For this reason, our primary goal was to investigate the MHS utilisation scenario in low- and middle-income countries (LMICs). It helped us pinpoint the general MHS utilisation influencing factors and their degree of impact on the outcome variable.

This study provides evidence on MHS utilisation in low- and middle-income countries. Therefore, this study might be helpful for policymakers in implementing different MHS programs to reduce maternal and child mortality. An all-out effort may reduce the problem to a bearable level and help us achieve the SDG of reducing the global maternal mortality ratio.

## Methods

### Data Sources and Sample Selection Procedures

This study considered nationally representative cross-sectional data from the Demographic and Health Survey (DHS) of 37 LMICs. The most recent DHS datsets conducted from 2015 and after were chosen based on data availability. In this study the considered countries were Bangladesh, Afghanistan, Albania, Armenia, Angola, Benin, Burundi, Cameroon, Gambia, Guinea, Guatemala, Haiti, India, Indonesia, Jordan, Cambodia, Liberia, Mali, Myanmar, Maldives, Malawi, Nigeria, Nepal, Papua New Guinea, Philippines, Pakistan, Rwanda, Sierra Leone, Senegal, Chad, Tajakistan, Timor-Leste, Tanzania, Uganda, South Africa, Zambia, and Zimbabwe. Data of women from the individual recode of the mensioned countreies were pooled and used for the final analysis. This analysis only included data for women of their reproductive age (15–49 years). In all 37 LMICs, the DHS followed the same standard procedures, and the survey data, detailed descriptions of sampling procedures, questionnaire validation and data collection methods are available at http://www.dhsprogram.com. A two-stage stratified sampling technique was used to select the respondents for the study. The enumeration areas (EAs) and households were randomly selected in the first and second stages. Out of a total of 1,296,281 women in pooled the dataset, after excluding all the missing and undefined observations, a final sample of 264,123 eligible women was chosen for the analysis.

### Outcome Variable

The outcome variable of our study was MHS utilisation. MHS utilisation was computed using 3 variables, i.e., ANC visits, SBA during the delivery of children (i.e., qualified doctor or nurse), and PNC during the postpartum period (within 42 days of childbirth). Women who had ≥4 ANC visits [16, 17], utilized SBA during their childbirth [18] and received PNC within 42 days of childbirth [19, 20] were considered as utilized MHS. Otherwise, if any women failed to meet any one of the mentioned 3 criteria, they were considered as not utilisation of MHS. The study outcome was reported as a binary variable with “utilisation of MHS” coded as ‘1’ (Yes), and “not utilisation of MHS” coded as ‘0’ (No).

### Explanatory Variables

The explanatory variables were considered as covariates based on their availability in the DHS dataset and extensive literature review [4, 9, 11–13, 19, 21]. The explanatory variables for this study were mothers’ place of residence (urban and rural), wealth index (poorest, poorer, middle, richer and richest), mother’s education (no education, primary, secondary and higher), husband/partner’s education (no education, primary, secondary and higher), mother’s age (15–24, 25–34, and 35–49 years), mother’s age at first birth (≤24, 25–34, and 35–49 years), family composition (≤4 and >4 members), number of living children (0–3, 4–6, and ≥7), mother’s working status (no and yes), mother’s healthcare decision-making autonomy (women alone, women and husband/others, and husband/others), and media exposure (no exposure, partial exposure, and full exposure). For husband/partner’s education, the women having husband or partner were considered for this study. Media exposure was created by considering three variables, which are the mother’s frequency of reading the newspaper (at least once a week = 1; else = 0), watching television (at least once a week = 1; else = 0), and listening to the radio (at least once a week = 1; else = 0). If a mother had all three of them, then it was classified as full exposure, if she had one or two of them, then it was classified as partial exposure, and if she had none of it, then it was classified as no exposure [9].

### Ethics Approval

Procedures and questionnaires for standard DHS surveys have been reviewed and approved by ICF Institutional Review Board (IRB). ICF IRB ensures that the survey complies with the U.S. Department of Health and Human Services regulations for the protection of human subjects, while the host country IRB ensures that the survey complies with laws and norms of the nation. All participants were informed of the purpose and procedure of the study prior to the survey. All participants completed the online informed consent before completing the survey.

### Statistical Analysis

Frequency and percentage were used to explore the background characteristics of the study variables in the selected LMICs. In addition, the weighted frequency was used to observe the prevalence of utilisation of MHS. This study cross-tabulated the distribution of utilisation of MHS across the explanatory variables, and the significant factors were identified using Pearson’s chi-square test of independence at a *p*-value of less than 0.05. Furthermore, univariate and multivariate binary logistic regression analysis was conducted to examine the critical explanatory variables’ association with the utilisation of MHS. The binary logistic regression model had all explanatory variables that were significant in Pearson’s chi-square test of independence, and multicollinearity was checked before model fit. The average VIF (Variance Inflation Factor) of all the variables was 1.617 (minimum = 1.041, maximum = 2.452), which was less than 10; thus, no sign of multicollinearity was found. The results were presented using crude odds ratios (COR) and adjusted odds ratios (AOR) at a 95% Confidence Interval (CI). To improve our findings’ generalisability, sample weight was used to correct for over and under-sampling, including the complex survey design. All the analyses were performed using SPSS 25 [22] and R software [23].

## Results

### Background Characteristics

The women’s background characteristics and the prevalence of utilisation of MHS were presented in Tables 1, 2, respectively. Overall, 89,122 (33.7%) women utilise MHS. Among the total women, the highest, 32,781 (12.4%), were from India, and the lowest, 1,171 (0.4%), were from South Africa. A total of 178,593 (67.6%) lived in rural areas, 61,798 (23.4%) were poorest, and 142,764 (54.1%) were not in paid employment. A total of 88,412 (33.5%) women had no education, 96,257 (36.4%) women’s husband/partner had secondary education, 131,409 (49.8%) were from the 25–34 years age group, and 194,285 (73.6%) women belonged to a large family (>4). However, 122,240 (46.3%) mothers were making healthcare decisions by discussing their husbands/others, and 134,442 (50.9%) had partial media exposure.

**TABLE 1:** Prevalence of utilisation of Maternal Healthcare services in the sample population across the 37 low and middle-income countries, 2015-2020.

Country	Frequency, n (%)	Utilisation of MHS		Prevalence of utilisation of MHS,% (95% CIs)	p–value
		No, n (%)	Yes, n (%)		
					<0.001
Bangladesh (2017–18)	4,927 (1.9)	3,299 (67.0)	1,628 (33.0)	31.8 (29.6–34.1)	
Afghanistan (2015)	19,161 (7.3)	17,194 (89.7)	1,967 (10.3)	11.7 (10.3–13.3)	
Albania (2017–18)	2,322 (0.9)	1,097 (47.2)	1,225 (52.8)	59.6 (55.9–63.1)	
Armenia (2015)	1,355 (0.5)	70 (5.2)	1,285 (94.8)	95.8 (94.5–96.9)	
Angola (2015–16)	5,731 (2.2)	4,927 (86.0)	804 (14.0)	14.4 (12.6–16.5)	
Benin (2017–18)	7,935 (3.0)	7,335 (92.4)	600 (7.6)	7.7 (6.7–8.8)	
Burundi (2016–17)	7,559 (2.9)	5,513 (72.9)	2,046 (27.1)	26.1 (24.7–27.6)	
Cameroon (2018)	4,845 (1.8)	2,746 (56.7)	2,099 (43.3)	42.9 (39.2–46.7)	
Gambia (2019–20)	4,982 (1.9)	1,774 (35.6)	3,208 (64.4)	66.2 (64.0–68.3)	
Guinea (2018)	5,049 (1.9)	4,029 (79.8)	1,020 (20.2)	21.2 (18.9–23.7)	
Guatemala (2014–15)	8,155 (3.1)	3,374 (41.4)	4,781 (58.6)	58.7 (56.2–61.2)	
Haiti (2016–17)	4,188 (1.6)	3,127 (74.7)	1,061 (25.3)	27.3 (24.8–30.0)	
India (2015–16)	32,781 (12.4)	19,607 (59.8)	13,174 (40.2)	43.3 (42.4–44.2)	
Indonesia (2017)	14,651 (5.5)	11,433 (78.0)	3,218 (22.0)	23.7 (22.5–25.0)	
Jordan (2017–18)	6,948 (2.6)	1,337 (19.2)	5,611 (80.8)	83.0 (81.4–84.5)	
Cambodia (2014–15)	5,555 (2.1)	4,731 (85.2)	824 (14.8)	16.2 (14.3–18.4)	
Liberia (2019–20)	2,762 (1.0)	853 (30.9)	1909 (69.1)	71.8 (68.7–74.8)	
Mali (2018)	5,836 (2.2)	4,377 (75.0)	1,459 (25.0)	24.7 (21.9–27.7)	
Myanmar (2015–16)	3,597 (1.4)	2093 (58.2)	1,504 (41.8)	43.1 (39.6–46.5)	
Maldives (2016–17)	2,371 (0.9)	1,708 (72.0)	663 (28.0)	35.4 (31.4–39.8)	
Malawi (2015–16)	10,899 (4.1)	7,958 (73.0)	2,941 (27.0)	27.1 (25.5–28.8)	
Nigeria (2018)	20,042 (7.6)	14,067 (70.2)	5,975 (29.8)	30.4 (28.5–32.3)	
Nepal (2016)	3,965 (1.5)	2,272 (57.3)	1,693 (42.7)	42.8 (39.5–46.1)	
Papua New Guinea (2016–18)	5,470 (2.1)	3,813 (69.7)	1,657 (30.3)	26.1 (23.7–28.7)	
Philippines (2017)	7,394 (2.8)	3,886 (52.6)	3,508 (47.4)	50.9 (48.3–53.6)	
Pakistan (2017–18)	8,149 (3.1)	5,188 (63.7)	2,961 (36.3)	38.6 (35.4–42.0)	
Rwanda (2019–20)	4,966 (1.9)	3,118 (62.8)	1848 (37.2)	37.5 (35.6–39.4)	
Sierra Leone (2019)	5,921 (2.2)	1,565 (26.4)	4,356 (73.6)	73.8 (71.4–76.0)	
Senegal (2019)	3,840 (1.5)	3,610 (94.0)	230 (6.0)	7.8 (6.3–9.7)	
Chad (2014–15)	9,533 (3.6)	8,880 (93.2)	653 (6.8)	7.9 (6.8–9.2)	
Tajikistan (2017)	4,089 (1.5)	1,620 (39.6)	2,469 (60.4)	59.5 (55.4–63.5)	
Timor Leste (2016)	4,785 (1.8)	2,965 (62.0)	1820 (38.0)	40.2 (36.8–43.8)	
Tanzania (2015–16)	5,731 (2.2)	4,622 (80.6)	1,109 (19.4)	20.4 (18.6–22.4)	
Uganda (2016)	8,186 (3.1)	5,352 (65.4)	2,834 (34.6)	35.1 (33.4–36.8)	
South Africa (2016)	1,171 (0.4)	271 (23.1)	900 (76.9)	74.4 (70.3–78.1)	
Zambia (2018)	5,291 (2.0)	2,772 (52.4)	2,519 (47.6)	48.4 (45.6–51.1)	
Zimbabwe (2015)	3,981 (1.5)	2,418 (60.7)	1,563 (39.3)	38.2 (35.9–40.7)	

**TABLE 2:** Prevalence of utilisation of Maternal Healthcare services in the sample population across the explanatory variables in 37 low and middle income countries, 2015-2020.

Explanatory variables	Frequency, n (%)	Utilisation of MHS		Prevalence of utilisation of MHS, % (95% CIs)	p–value
		No, n (%)	Yes, n (%)		
MHS utilisation					
No	175,001 (66.3)				
Yes	89,122 (33.7)			34.7 (34.2-35.2)	
Place of residence					<0.001
Urban	85,530 (32.4)	44,622 (52.2)	40,908 (47.8)	50.7 (49.8–51.6)	
Rural	178,593 (67.6)	130,379 (73.0)	48,214 (27.0)	26.7 (26.1–27.2)	
Wealth index					<0.001
Poorest	61,798 (23.4)	48,752 (78.9)	13,046 (21.1)	20.1 (19.4–20.7)	
Poorer	57,809 (21.9)	42,188 (73.0)	15,621 (27.0)	26.8 (26.2–27.5)	
Middle	53,350 (20.2)	35,383 (66.3)	17,967 (33.7)	33.7 (33.0–34.5)	
Richer	48,850 (18.5)	29,072 (59.5)	19,778 (40.5)	41.9 (41.0–42.8)	
Richest	42,316 (16.0)	19,606 (46.3)	22,710 (53.7)	55.3 (54.2–56.4)	
Education					<0.001
No education	88,412 (33.5)	73,150 (82.7)	15,262 (17.3)	17.2 (16.6–17.8)	
Primary	70,719 (26.8)	49,859 (70.5)	20,860 (29.5)	29.7 (29.1–30.3)	
Secondary	82,168 (31.1)	44,290 (53.9)	37,878 (46.1)	47.5 (46.9–48.1)	
Higher	22,824 (8.6)	7,702 (33.7)	15,122 (66.3)	69.2 (68.1–70.2)	
Education husband/partner					<0.001
No education	69,611 (26.4)	57,162 (82.1)	12,449 (17.9)	17.8 (17.1–18.5)	
Primary	68,521 (25.9)	49,509 (72.3)	19,012 (27.7)	27.8 (27.2–28.4)	
Secondary	96,257 (36.4)	55,816 (58.0)	40,441 (42.0)	43.5 (42.9–44.2)	
Higher	29,734 (11.3)	12,514 (42.1)	17,220 (57.9)	60.7 (59.8–61.7)	
Mother’s age					<0.001
15–24	71,084 (26.9)	48,145 (67.7)	22,939 (32.3)	33.3 (32.6–34.0)	
25–34	131,409 (49.8)	84,865 (64.6)	46,544 (35.4)	36.5 (35.9–37.1)	
≥35	61,630 (23.3)	41,991 (68.1)	19,639 (31.9)	32.4 (31.8–33.1)	
Mother’s age at first birth					<0.001
≤24	226,653 (85.8)	155,422 (68.6)	71,231 (31.4)	32.3 (31.8–32.9)	
25–34	35,954 (13.6)	18,886 (52.5)	17,068 (47.5)	48.8 (48.0–49.7)	
≥35	1,516 (0.6)	693 (45.7)	823 (54.3)	57.0 (53.7–60.3)	
Family composition					<0.001
≤4	69,838 (26.4)	42,732 (61.2)	27,106 (38.8)	39.7 (39.1–40.4)	
>4	194,285 (73.6)	132,269 (68.1)	62,016 (31.9)	32.8 (32.2–33.3)	
Number of living children					<0.001
≤3	174,603 (66.1)	108,245 (62.0)	66,358 (38.0)	39.1 (38.6–39.6)	
4–6	70,307 (26.6)	51,339 (73.0)	18,968 (27.0)	27.4 (26.7–28.0)	
≥7	19,213 (7.3)	15,417 (80.2)	3,796 (19.8)	19.5 (18.7–20.5)	
Mother’s working status					<0.001
No	142,764 (54.1)	94,071 (65.9)	48,693 (34.1)	35.5 (34.9–36.1)	
Yes	121,359 (45.9)	80,930 (66.7)	40,429 (33.3)	33.8 (33.2–34.4)	
Mother’s healthcare decision maker					<0.001
Women alone	46,193 (17.5)	28,909 (62.6)	17,284 (37.4)	38.1 (37.2–38.9)	
Women & husband/others	122,240 (46.3)	75,002 (61.4)	47,238 (38.6)	39.9 (39.3–40.5)	
Husband/others	95,690 (36.2)	71,090 (74.3)	24,600 (25.7)	26.5 (25.8–27.2)	
Media exposure					<0.001
No exposure	119,560 (45.3)	91,965 (76.9)	27,595 (23.1)	23.1 (22.6–23.7)	
Partial exposure	134,442 (50.9)	79,043 (58.8)	55,399 (41.2)	42.1 (41.5–42.8)	
Full exposure	10,121 (3.8)	3,993 (39.5)	6,128 (60.5)	62.5 (60.9–64.0)	

### Prevalence of Utilisation of MHS

Overall, the prevalence of utilisation of MHS was 34.7% (95% CI: 34.2%-35.2%) among the LMICs. The prevalence of utilisation of MHS varied among countries from 7.7% (95% CI: 6.7%–8.8%) in Benin to 95.8% (95% CI: 94.5%–96.9%) in Armenia (Table 1). Among all countries, Albania, Armenia, Gambia, Guatemala, Jordan, Liberia, Philippines, Sierra Leone, and Tajikistan had more than 50% prevalence of utilisation of MHS. The prevalence of utilisation of MHS was higher among women in urban areas (50.7%, 95% CI: 49.8%–51.6%), with higher education (69.2%, 95% CI: 68.1%–70.2%), in the 25–34 years age group (36.5%, 95% CI: 35.9%–37.1%), and within the richest household segment (55.3%, 95% CI: 54.2%–56.4%). The prevalence of utilisation of MHS among the women whose husband/partners had higher education (60.7%, 95% CI: 59.8%–61.7%) and had comparatively small families (39.7%, 95% CI: 39.1%–40.4%). The prevalence of utilisation of MHS among the women who are either able to make their own healthcare decisions or after consulting with their husband/others was 38.1% (95% CI: 37.2%–38.9%) and 39.9% (95%CI: 39.3%–40.5%), respectively. The prevalence of utilisation of MHS was also higher among the women who had full access to media (62.5%, 95% CI: 60.9%–64.0%) (Table 2).

### Multivariate Analysis

**FIGURE 1:**
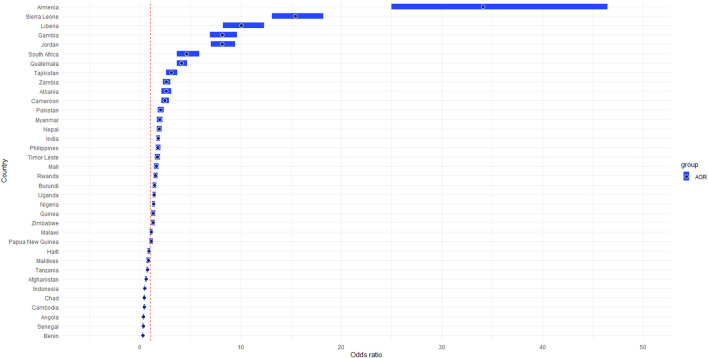
Forest plot of the adjusted odds ratio for 37 low and middle-income countries, 2015-2020.

Figure 1 represents the adjusted odds ratio (AOR) of all 36 countries compared with Bangladesh. All the countries had a significant difference in utilisation of MHS except Papua New Guinea (*p* = 0.078). The odds of the utilisation of MHS were highest in Armenia (AOR = 34.08, 95% CI = 24.99–46.47), while the lowest in Benin (AOR = 0.28, 95% CI = 0.24–0.33), compared to Bangladesh.

Table 3 presents the factors associated with the utilisation of MHS. Mothers residing in the urban area (AOR = 1.56, 95% CI = 1.49–1.64) were significantly more likely to utilise MHS than those of rural areas. The odds of the utilisation of MHS were 1.20 times (AOR = 1.20, 95% CI: 1.15–1.25) higher among poorer women, 1.35 times (AOR = 1.35, 95% CI: 1.29–1.41) higher among middle-class women, 1.45 times (AOR = 1.45, 95% CI: 1.37–1.52) higher among richer women, and 1.56 times (AOR = 1.56, 95% CI: 1.49–1.67) higher among richest women compared with poorest women. Mother’s primary education (AOR = 1.58, 95% CI = 1.52–1.65), secondary education (AOR = 2.28, 95% CI = 2.18–2.38) and higher education (AOR = 3.77, 95% CI = 3.53–4.03) had a significantly higher chance of utilising MHS compared to the non-educated mothers. The odds of the utilisation of MHS were 1.08 times (AOR = 1.08, 95% CI: 1.05–1.11) higher among those aged 25–34 years and 1.19 times (AOR = 1.19, 95% CI: 1.14–1.25) higher among those aged 35–49 years compared with women aged ≤24 years. Similarly, mothers whose age at their first birth at 25–34 years were 1.12 times (AOR = 1.12, 95% CI = 1.08–1.17), and between 35–49 years were 1.56 times (AOR = 1.56, 95% CI = 1.36–1.80) were more likely to utilise MHS than mothers whose age at first birth was ≤24 years. Mothers’ healthcare decisions after consulting with their husbands/others (AOR = 1.19, 95% CI = 1.15–1.24) were significantly more likely to utilise MHS than mothers who took their healthcare decisions by themselves. In addition, women with partial media exposure (AOR = 1.29, 95% CI = 1.29–1.33) and full media exposure (AOR = 1.70, 95% CI = 1.58–1.83) were more likely to utilise MHSs than mothers without any media exposure. On the other hand, mothers living in the larger families (>4) (AOR = 0.93, 95% CI = 0.91–0.96) were less likely to utilise MHS than mothers living in the small families (≤4). However, mother’s healthcare decisions were made by their husbands/others (AOR = 0.92, 95% CI = 0.88–0.97) were less likely to utilise MHS than mothers who took their healthcare decisions by themselves. Mothers having 4–6 living children (AOR = 0.88, 95% CI = 0.85–0.91) and ≥7 living children (AOR = 0.72, 95% CI = 0.68–0.77) were less likely to utilise MHS than mothers having ≤3 living children.

**TABLE 3:** Factors associated with utilisation of Maternal Healthcare services among reproductive-aged (15–49 years) women in 37 low- and middle-income countries, 2015-2020.

	COR (95% CIs)	*p*-value	AOR (95% CIs)	*p*-value
Place of residence				
Rural	(Reference)		(Reference)	
Urban	2.83 (2.71–2.96)	<0.001	1.56 (1.49–1.64)	<0.001
Wealth index				
Poorest	(Reference)		(Reference)	
Poorer	1.46 (1.40–1.52)	<0.001	1.20 (1.15–1.25)	<0.001
Middle	2.03 (1.94–2.13)	<0.001	1.35 (1.29–1.41)	<0.001
Richer	2.87 (2.73–3.02)	<0.001	1.45 (1.37–1.52)	<0.001
Richest	4.93 (4.65–5.23)	<0.001	1.56 (1.46–1.67)	<0.001
Education				
No education	(Reference)		(Reference)	
Primary	2.03 (1.95–2.12)	<0.001	1.58 (1.52–1.65)	<0.001
Secondary	4.36 (4.17–4.56)	<0.001	2.28 (2.18–2.38)	<0.001
Higher	10.81 (10.16–11.51)	<0.001	3.77 (3.53–4.03)	<0.001
Education husband/partner				
No education	(Reference)		(Reference)	
Primary	1.78 (1.69–1.87)	<0.001	1.13 (1.08–1.18)	<0.001
Secondary	3.56 (3.39–3.74)	<0.001	1.43 (1.36–1.50)	<0.001
Higher	7.14 (6.73–7.59)	<0.001	1.68 (1.59–1.79)	<0.001
Mother’s age				
15–24	(Reference)		(Reference)	
25–34	1.15 (1.12–1.18)	<0.001	1.08 (1.05–1.11)	<0.001
≥35	0.96 (0.93–0.99)	0.001	1.19 (1.14–1.25)	<0.001
Mother’s age at first birth				
≤24	(Reference)		(Reference)	
25–34	2.00 (1.93–2.07)	<0.001	1.12 (1.08–1.17)	<0.001
≥35	2.78 (2.43–3.18)	<0.001	1.56 (1.36–1.80)	<0.001
Family composition				
≤4	(Reference)		(Reference)	
>4	0.74 (0.72–0.76)	<0.001	0.93 (0.91–0.96)	<0.001
Number of living children				
≤3	(Reference)		(Reference)	
4–6	0.59 (0.57–0.60)	<0.001	0.88 (0.85–0.91)	<0.001
≥7	0.38 (0.36–0.40)	<0.001	0.72 (0.68–0.77)	<0.001
Mother’s working status				
No	(Reference)		(Reference)	
Yes	0.93 (0.90–0.96)	<0.001	1.00 (0.97–1.03)	0.803
Mother’s healthcare decision maker				
Women alone	(Reference)		(Reference)	
Women & husband/others	1.08 (1.04–1.12)	<0.001	1.19 (1.15–1.24)	<0.001
Husband/others	0.59 (0.56–0.62)	<0.001	0.92 (0.88–0.97)	<0.001
Media exposure				
No exposure	(Reference)		(Reference)	
Partial exposure	2.42 (2.24–2.50)	<0.001	1.29 (1.25–1.33)	<0.001
Full exposure	5.53 (5.15–5.93)	<0.001	1.70 (1.58–1.83)	<0.001

## Discussion

This study aimed to identify the prevalence and risk factors of the utilisation of MHS in 37 LMICs. Despite various nationwide work on maternal healthcare utilisation, this study is an extensive and worldwide work of LMICs. This study mainly concentrated on the factor wise prevalence of utilisation of MHS and attempted to figure out the significant determinants.

The study revealed that the prevalence varies significantly across the countries, indicating diverse levels of maternal healthcare utilisation. Countries like Armenia (95.8%), Jordan (83.0%), and Sierra Leone (73.8%) have notably high maternal healthcare utilisation rates. This could be attributed to factors such as strong healthcare infrastructure, awareness campaigns, or cultural emphasis on maternal health, better development indicators, education levels and national health policies [24–26]. Conversely, countries like Senegal (7.8%), Chad (7.9%), and Benin (7.7%) exhibit much lower utilisation prevalence. Limited access to healthcare facilities, cultural barriers, low awareness, gender roles, poverty, might contribute to these lower rates along with the regional patterns, where people struggle to receive adequate healthcare services, impacting utilisation of MHS [2, 10]. Facility assessment surveys conducted in developing countries such as Bangladesh and Nepal found that the availability of staff and guideline, medical equipment, diagnostic facility and medicine is comparatively low in rural areas compared to the urban areas highlighting the lower prevalence of utilisation of MHS found in Bangaldesh (31.8%) and Nepal (42.8%) [27].

The result of the present study showed that place of residence was significantly associated with the utilisation of MHS. Urban women were more likely to utilise MHS compared to their rural counterparts. This result is consistent with the findings of similar studies conducted in South Asian countries and a country in Sub-Saharan Africa [9, 13, 14]. The possible reason might be that urban women received various benefits such as more knowledge, awareness, and easy accessibility to government and private health service facilities. Besides, urban women typically gave more attention to their education which might played a positive role in this regard as well [9, 21]. It is indisputable that shorter distances, paved infrastructure and public transit can motivate urban women to seek more healthcare services than women in rural regions [13]. However, other research has highlighted a link between miserable maternal healthcare outcomes and urban areas, particularly in poor slum communities near major cities [28].

The wealth index was identified as another significant influencing factor positively connected with the utilisation of MHS. The result of this study revealed a progressive rise in utilisation of MHS from poorest to richest, which was consistent with findings of studies performed in LMICs such as India, Indonesia, Uganda and Nigeria [4, 9, 11, 13, 19, 21]. This can be explained by the fact that financially well-off women have higher access to healthcare resources and prefer a better hospital facility [21]. Some other factors often intersect with wealth, influencing healthcare-seeking behaviors. For instance, individuals with higher education levels and residing in urban areas tend to have greater access to information about healthcare services through media exposure, which can contribute to improved healthcare utilisation [13, 19, 21]. It also points out the household’s capability to meet healthcare utilisation costs [11], where the poor women are burdened financially in providing their family’s maternal demands [13].

Women’s education was significantly positively associated with the utilisation of MHS. This result clearly indicated that the level of education increases the likelihood of utilisation of MHS and is consistent with the previous study findings [9, 11–13, 19, 21]. These findings underlined the significance of women’s education in improving maternity care services. The possible reason behind this could be that educated women have greater awareness about health information and are more aware of the harmful effects of failing to seek maternity care. Furthermore, educated women have decision-making capacities regarding healthcare [29, 30]. Alternatively, lack of education can hinder women from being aware of the critical obstetric care services, reducing their understanding of the necessity to seek risk-appropriate medical care [4, 11].

Besides women’s education, the findings showed that the husband/partner’s education also had a significant positive impact on the utilisation of MHS. The results showed that the odds of receiving utilisation of MHS increased as the husband/partner’s education levels had increased successively. This result is compatible with the findings of a study in Nigeria [12]. This could possibly be that well-educated husband/partners are able to understand the health information more easily, and their involvement in women’s health issues improves the latter’s exposure to reproductive and maternal healthcare utilisation [31–33]. Furthermore, studies have shown that when husband/partners agree on prioritising a woman’s healthcare demand, it is handled efficiently and quickly [12]. Both women’s education and the education level of their husband/partners play integral roles in maternal healthcare utilisation. Educated women are better equipped to make informed health decisions, while well-educated husband/partners contribute to creating a supportive environment that encourages timely access to maternal health services [12, 32, 33].

The mother’s age was significantly associated with the utilisaton of MHS. This study demonstrated that older women were more likely to utilise MHS than younger women, which is similar to the findings of a study conducted in Indonesia [13]. This could be because younger women have restrictions on their controlling and managing capability due to insufficient knowledge about maternal healthcare services and age maturity, whereas older women have better health expertise and are more aware of the negative consequences of maternity issues [13, 34]. Another fact is that even though DHS consider reproductive women of age 15–49 years. A study among the adolescent sexual and reproductive health (ASRH) found that, in Bangladesh between 1996 and 2011 the contraceptive use among women aged 10–49 years increased from 49% to 61%, supporting the fact that in some LMIC countries women become reproductive before the age of 15 years [35].

Besides, the mother’s age at first birth had a significant positive relationship with the utilisation of MHS. The results revealed that mothers who had their first birth at an older age were more likely to utilise MHS than those who had their first birth at an earlier age, which is consistent with the results found in Nigeria [19]. This might be due to younger women often experiencing confusing feelings and emotional fragility when it comes to their new role as mothers [34]. Based on previous research, another reason could be that premarital and unplanned pregnancies are more common among teenagers than the older women, and unwanted and premarital births lead to poorer maternal healthcare where older mothers barely face these consequences and are more conscious about maternal complications [36, 37]. Both the mother’s age and the age at first birth are vital factors influencing the utilisation of MHS simultaneously. In a nutshell, the age maturity level is crucial in pregnancy-related decision-making, especially at a young age; therefore, family and health workers should provide a great deal of attention [13].

The family composition had a significant negative association with the utilisaton of MHS. The result indicated that women from larger families were less likely to receive the utilisation of MHS than women with a family size of four or less. This result is consistent with the findings revealed in Bangladesh and China [38, 39]. This could be due to too many responsibilities on their shoulders, and women from big families underutilise available healthcare facilities. Some extended families also lead to resource limitations, negatively impacting healthcare consumption [39]. On the other hand, smaller families boost mothers’ food consumption and other necessities, resulting in proper healthcare utilisation [38].

The current study also showed that the number of living children was negatively associated with the utilisation of MHS. The outcome showed that women with a number of living children of 4–6, and 7 or more had lower odds of using utilisaton of MHS than women with 3 or fewer children. This result is related to the findings of a Chinese study [38]. The possible reason could be that the resource challenge amongst the family members increases when there are many children and belong to the poorer quintile. As a result, both mother and children may not afford food and health-related expenses [38]. However, this result is inconsistent with the findings in Sub-Saharan African countries, which identified a positive association between the number of children with maternal healthcare [10]. This might be because women are more aware of the significance of greater maternal arrangement as they enter motherhood [13]. The family composition, specifically belonging to a larger family, and having a higher number of living children both negatively impact the utilisation of maternal healthcare services. These findings highlight the challenges that women from larger families or those with more children might face in accessing healthcare due to resource limitations and increased responsibilities. It also emphasizes the need for tailored strategies and interventions to ensure that women from such backgrounds receive adequate maternal healthcare support [13, 38].

Mother’s healthcare decision-making autonomy was significantly related to the utilisation of MHS in the present study. The result showed that the combined decision of women and husbands or others was more likely to receive MHS compared to women alone or decisions made by only husbands or others, which is related to the study done in Ghana [40]. The possible explanation could be that women alone sometimes face obstetric difficulties and cannot always take the proper decision during the vulnerable period of motherhood. Still, their husband or other family members’ mental and emotional support throughout the time and their combined decision can lead to improved utilisation of MHS [24, 40]. In addition, mother’s ability to actively participate in healthcare decision-making is closely intertwined with her household’s economic status, level of education, and exposure to healthcare-related information. These factors collectively shape her capacity to access and utilise maternal healthcare services effectively [11, 13, 40].

Media exposure had a significant impact on the utilisation of MHS. The present result revealed that those who were exposed to full media or partial media were more likely to utilise MHS compared to the women who had no media exposure, which is compatible with the findings performed in India and Nigeria [9, 12, 21]. The possible argument could be access to mass media, notably newspapers, radio and television, promotes the acquisition of healthcare knowledge and raises public awareness of health issues [9]. Yet, an international study found that reproductive women in the lowest quintile had considerably less possibility of reporting consistent media exposure than women in the wealthiest quintile [41]. Educational activities broadcast over the public network must address the poorest reproductive illiterate women from socially deprived communities residing in rural regions [12]. Media coverage affects maternal healthcare use and is linked to wealth index, education and family composition. To guarantee that all women can obtain maternity healthcare information, media access must be addressed, especially among economically deprived and rural populations.

### Strengths and Limitations

The most vital point of the current study is the pooled population-based survey considering 37 LMICs, using the most recent DHS data of 2015 or later to examine the prevalence and related factors of the utilisation of MHS. Therefore, the findings of this inquiry can be generalised to the target demographics of the selected countries. Also, the study utilised a bigger sample size and better-quality data, which reduced the potential risk of sampling and measurement bias significantly. However, there exist some drawbacks to this study. Firstly, the causation cannot be explained by the cross-sectional design of the DHS survey, which estimated all variables during the same timeframe. Second, the survey was built on the mothers’ self-reported health-related information, which allowed for the risk of recall bias. Third, some LMICs were excluded from the analysis due to a lack of up-to-date data from the DHS survey, and some established factors reported to be relevant in previous studies, such as religion and distance to healthcare facilities, were also eliminated from the study due at unavailability of information for the selected countries.

### Conclusion

The study analysed the utilisation of MHS considering the ANC, SBA, and PNC among reproductive-aged women 15–49 years old living in LMICs and indicated that several factors were significantly associated with utilisation of MHS. The study concluded that urban women, the richest quintile, had higher education, highly educated husband/partners, higher age of the women had first birth at 35 years or more, had women and husbands/others’ joint healthcare decisions and were fully exposed to media were more likely to get utilisation of MHS. Alternatively, a larger family with 7 or more children were negatively associated with utilisation of MHS. The varied prevalence of utilisation of MHS and substantial differences in numerous dimensions among mothers aged 15–49 years in the selected countries revealed the obstacles that reproductive-aged women experience in utilising MHS. This study offers decision-makers with some valuable insights to improve the utilisation of MHS policies and strategies for women of reproductive age. To maximise utilisation of maternal healtcare among 15–49 years-aged mothers, national policy should focus on service equity, accessibility and efficient implementation of MHS. A massive public awareness campaign targeting reproductive mothers about the importance of utilisation of MHS may help them more about the issue of MHS. Policymakers and public and private healthcare providers at all levels should be concerned about the proper administration of tailored initiatives and programs to enhance the utilisation of MHS among women.
